# Conserved and distinct morphological aspects of the salivary glands of sand fly vectors of leishmaniasis: an anatomical and ultrastructural study

**DOI:** 10.1186/s13071-020-04311-y

**Published:** 2020-09-03

**Authors:** Rafael Nacif-Pimenta, Luciana C. Pinto, Vera Volfova, Petr Volf, Paulo F. P. Pimenta, Nagila F. C. Secundino

**Affiliations:** 1 Laboratory of Medical Entomology, Institute René Rachou, Foundation Oswaldo Cruz, Fiocruz-MG, Belo Horizonte, Brazil; 2 Department of Parasitology, Charles University, Prague, Czech Republic

**Keywords:** Secretory cell population, Ultrastructure, Lectin binding, Sand fly vectors

## Abstract

**Background:**

Sand flies are vectors of *Leishmania* spp., the causative agents of leishmaniasis in vertebrates, including man. The sand fly saliva contains powerful pharmacologically active substances that prevent hemostasis and enhance *Leishmania* spp. infections. On the other hand, salivary proteins can protect vaccinated mice challenged with parasites. Therefore, sand fly salivary proteins are relevant for the epidemiology of leishmaniasis and can be a potential target for a vaccine against leishmaniasis. Despite this, studies on sand fly salivary glands (SGs) are limited.

**Methods:**

The present study analyzes, in detail, the morphology, anatomy and ultrastructure of the SGs of sand fly vectors of the genera *Lutzomyia* and *Phlebotomus*. We used histology, transmission and scanning electron microscopy and lectin labeling associated with confocal laser microscopy.

**Results:**

The SGs have conserved and distinct morphological aspects according to the distinct sand fly species. Each SG has a single rounded lobe constituting of *c.*100–120 secretory cells. The SG secretory cells, according to their ultrastructure and lectin binding, were classified into five different subpopulations, which may differ in secretory pathways.

**Conclusions:**

To the best of our knowledge, these morphological details of sand fly salivary glands are described for the first time. Further studies are necessary to better understand the role of these different cell types and better relate them with the production and secretion of the saliva substances, which has a fundamental role in the interaction of the sand fly vectors with *Leishmania*.
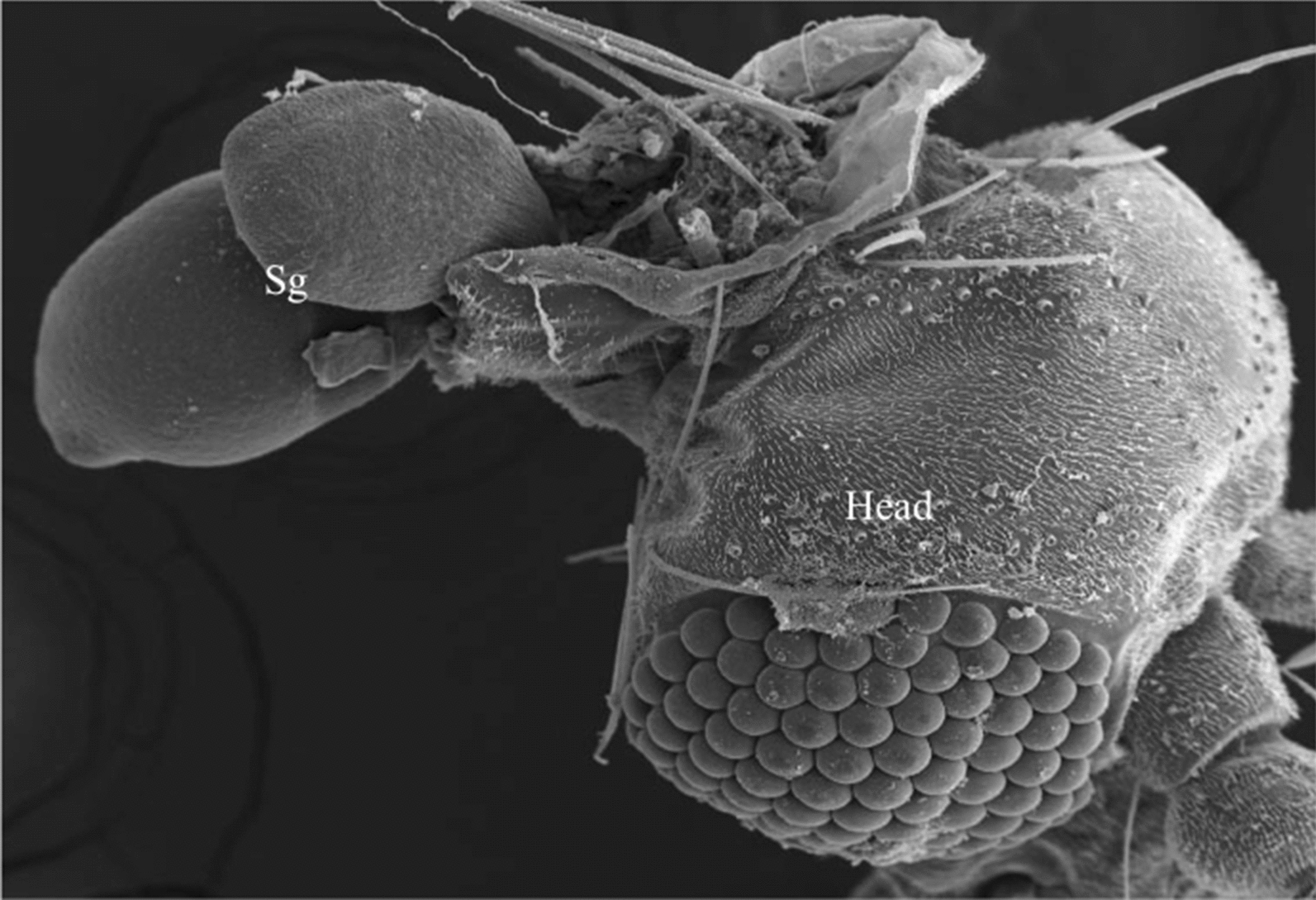

## Background

Female sand flies (Diptera: Psychodidae) are blood-feeding insects and the main vectors of the parasite *Leishmania* (Ross, 1903), the causative agent of leishmaniasis and a neglected tropical disease with worldwide distribution. Over 90 sand fly species are known to transmit *Leishmania* parasites affecting people in 98 countries including 18 in the Americas [1]. The sand fly genus *Phlebotomus* (Loew, 1845) is responsible for Old World transmission and the genus *Lutzomyia* (France, 1924) for America transmission [2]. About 31 species of *Leishmania* parasites have been identified to date to be parasites of mammals and 20 species are pathogenic for humans (see [3] for a review).

The bite of infected female sand flies transmits *Leishmania* parasites. During the process of blood-feeding, the sand fly cuts the host skin and blood vessels with the proboscis. The host defends from the skin injury by activating hemostasis, inflammation and immunity, such as anti-salivary antibody production [4]. The sand fly uses the saliva to counter these defenses to accomplish a successful blood meal *via* powerful pharmacologically active substances; hence, the saliva plays an essential role in infection establishment. In many laboratory models, *Leishmania* spp. co-inoculated with saliva or saliva proteins show a higher infection rate than inoculation of parasites alone [5, 6]. The co-evolutionary parasite-vector relationship allows the *Leishmania* parasite to use the vector’s saliva to its advantage. The proteins described in the sand fly saliva facilitate entry and survival of the parasite [7, 8].

During an insect’s life, the saliva will lubricate, solubilize and help to digest the nectar or blood [9, 10]. Indeed, several different types of molecules with anticlotting, antiplatelet, vasodilatory and immunosuppressive activities have been described and characterized in the sand fly saliva [4, 6, 8, 10, 11]. Interestingly, the complexity of the saliva composition is closely related to the efficient ability of the sand fly’s blood taking from a vertebrate host, since male (non-hematophagous) sand flies possess 30 times lower protein concentration in their saliva when compared to female sand flies [12].

In contrast to multiple studies on the role of the sand fly saliva, there are very few detailed studies on SG morphology. Adler & Theodor [13] and Perfiliev [14] described the SGs of three species of *Phlebotomus* as a paired organ composed by two saccular structures (lobes) with a lumen surrounded by a single epithelial tissue. More recently, Abdel-Badei et al. [15] showed that the sizes of the SG lobes differ in *Phlebotomus papatasi* but are equal in *P. langeroni*. Similarly, Lestinova et al. [6] found that *P. duboscqi* possess heterogenous size SG lobes (corroborating results of Adler & Theodor [13]) while *L. longipalpis* has homogenous gland lobes, as previously described by Secundino & Pimenta [16].

The present study focuses on analyzing in detail the morphology, microanatomy and ultrastructure of the SGs of some important species of sand fly vectors of leishmaniasis of the genera *Lutzomyia* and *Phlebotomus*. Using morphological techniques such as scanning electron microscopy (SEM) and transmission electron microscopy (TEM) associated with lectin labeling visualized by laser scanning confocal microscopy (LSM), we showed conserved morpho-structural aspects among the sand fly SGs as well as the presence of different subpopulations of secretory cells which can be distinct according to the sand fly species.

## Methods

### Sand flies

*Lutzomyia longipalpis* and *L. migonei* were reared in closed colonies at the Laboratory of Medical Entomology, Fiocruz-MG, Brazil. *Phlebotomus* sand flies (*P. duboscqi*, *P. halepensis* and *P. sergenti*) were raised at the insectary of the Department of Parasitology, Charles University, Czech Republic. The sand fly colonies were maintained in the insectaries according to conditions described by Killick-Kendrick et al. [17, 18] and Volf & Volfova [19].

### SG dissection

Four-day-old female sand flies maintained only on 50% sucrose *ad libidum* (non-blood-fed) were anesthetized on ice or in a refrigerator and dissected over a glass slide in cold phosphate-buffered saline (PBS), pH 7.2. The sand fly head was slowly detached from the thorax until its complete separation from the body, exposing the attached SG. Carefully, the heads with the attached SGs were fixed directly over the slide for 3 min and transferred to Eppendorf tubes containing the fixative. This method allowed us to work with a larger sample instead of handling minuscule SGs, facilitating the use of several morphological methods and several samples. Sample sizes of at least 10 SGs for each sand fly species were dissected for the experiments, which were repeated 3 times.

### SG fixation

The dissected SGs were fixed 2.5% glutaraldehyde solution in 0.1 M cacodylate buffer for TEM and SEM, or in a 4% formaldehyde solution in PBS for lectin labeling. The samples were fixed inside Eppendorf tubes with the fixative at room temperature for 2 h and stored, until use, in PBS at 4 °C [20].

### SEM, TEM and histology

The glutaraldehyde-fixed SGs were washed three times in PBS and post-fixed in 1% osmium tetroxide solution plus 0.8% potassium ferrycianide with 0.1 M cacodylate buffer at pH 7.2 [20]. Some samples were dehydrated in a graded acetone series, critical-point dried using liquid CO_2_, mounted on stubs, and coated with gold using a sputter-coater to be analyzed under SEM [16]. Samples dedicated for TEM and histology examination, were embedded in Epon resin as described in Nacif-Pimenta et al. [21]. Ultra-thin sections (400 Å) were obtained in an ultramicrotome, contrasted with uranyl acetate and lead citrate, and washed in distilled water to be analyzed under TEM. Thin histological sections (1 µm) were mounted onto glass slides, stained with 0.1% toluidine blue solution to be analyzed under light microscopy

### Counting the SG cell number

Formaldehyde-fixed SGs were washed several times in PBS and incubated with a thick drop of the fluorescent nuclear marker DAPI (4,6 diamidino-2-phenylindole; Sigma-Aldrich, St. Louis, USA) for 10 min. Whole SGs were placed in concave microscope slides, mounted with Vectashield® medium (Vector Laboratories, Burlingame, USA) to avoid fluorescence fading, and photographed under a fluorescence microscope. The number of cells present in each SG was assessed by counting the fluorescent nuclei in a series of photomicrographs obtained by taking images with sequential focuses of the entire organ.

### Lectin binding

Formaldehyde-fixed SGs were washed several times with PBS and incubated in the dark for 1 h with the following FITC-fluorescent lectins (1:50 v/v): Con A (*Canavalia ensiformis*), RCA (*Ricinus communis*), WGA (*Triticum vulgaris*), HPA (*Helix pomatia*), UEA (*Ulex europaeus*) (all from Sigma-Aldrich). After incubation, the samples were washed several times in PBS, mounted with Vectashield® (Vector Laboratories) and observed under a fluorescence microscope (Olympus BX51 with camera DP72; Olympus Co., Shinjuku, Japan) or a Zeiss LSM 510 laser scanning microscope (Carl Zeiss AG, Oberkochen, Germany).

## Results

### Electron microscopy and histology of SGs

**Fig. 1:**
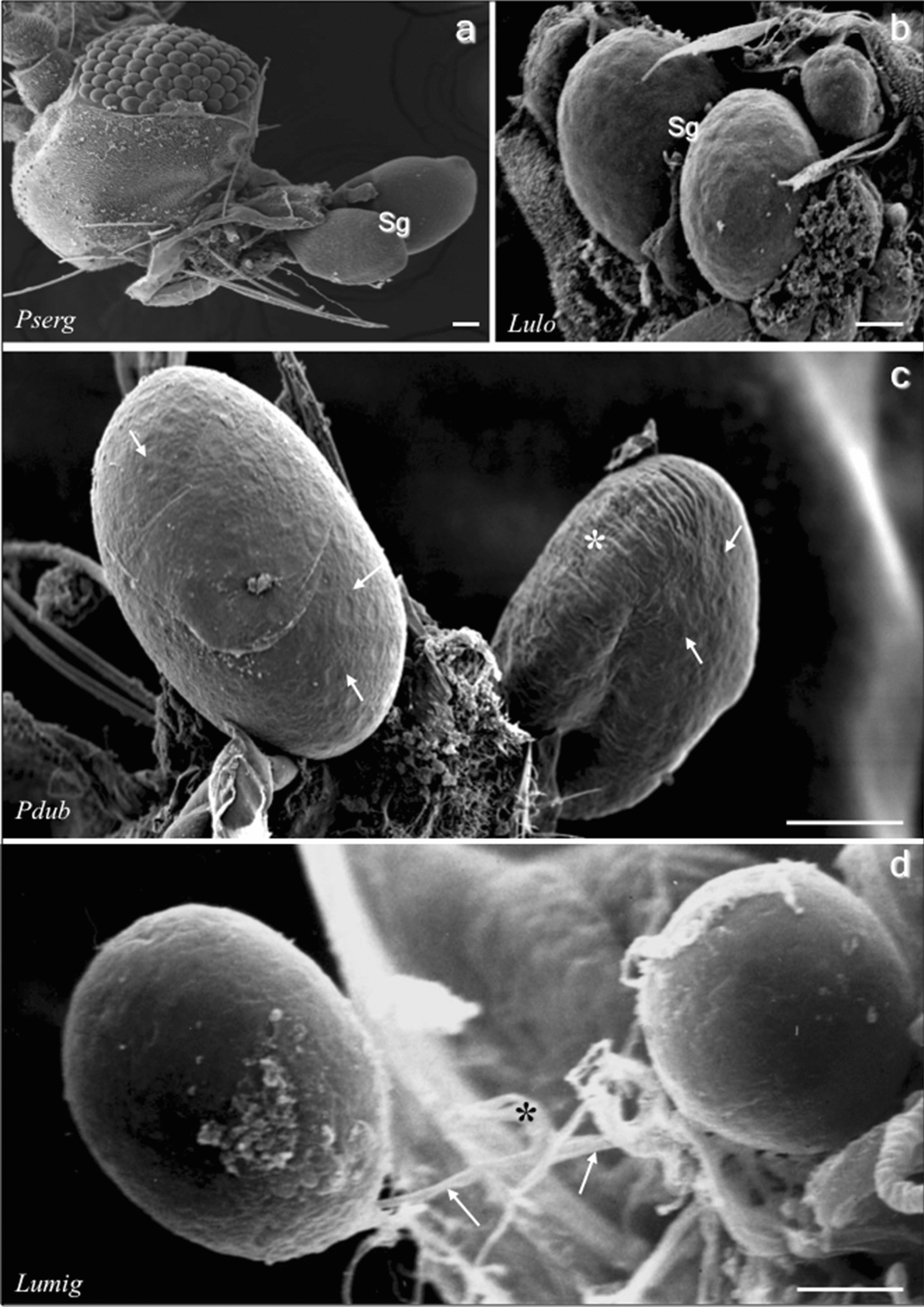
SEM micrographs of sand fly SGs of *P. sergenti* (**a**), *Lu. longipalpis* (**b**), *P. duboscqi* (**c**) and *Lu. migonei* (**d**). Sandfly SGs are composed of two rounded lobes; lobe surfaces are turgid (**c**, also see Fig. 2**b**) showing marks of the secretory cells (arrows) or slack surface (asterisk in **c**). Panel **d** shows small ducts (arrows) linking the SG lobes to the common salivary duct (asterisk). *Abbreviations*: *Pserg*, *P. sergenti*; *Lulo*, *Lu. longipalpis*; *Pdub*, *P. duboscqi*; *Lumig*, *Lu. migonei. Scale-bars*: **a**, **b**, 50 µm; **c**, **d**, 100 µm

**Fig. 2:**
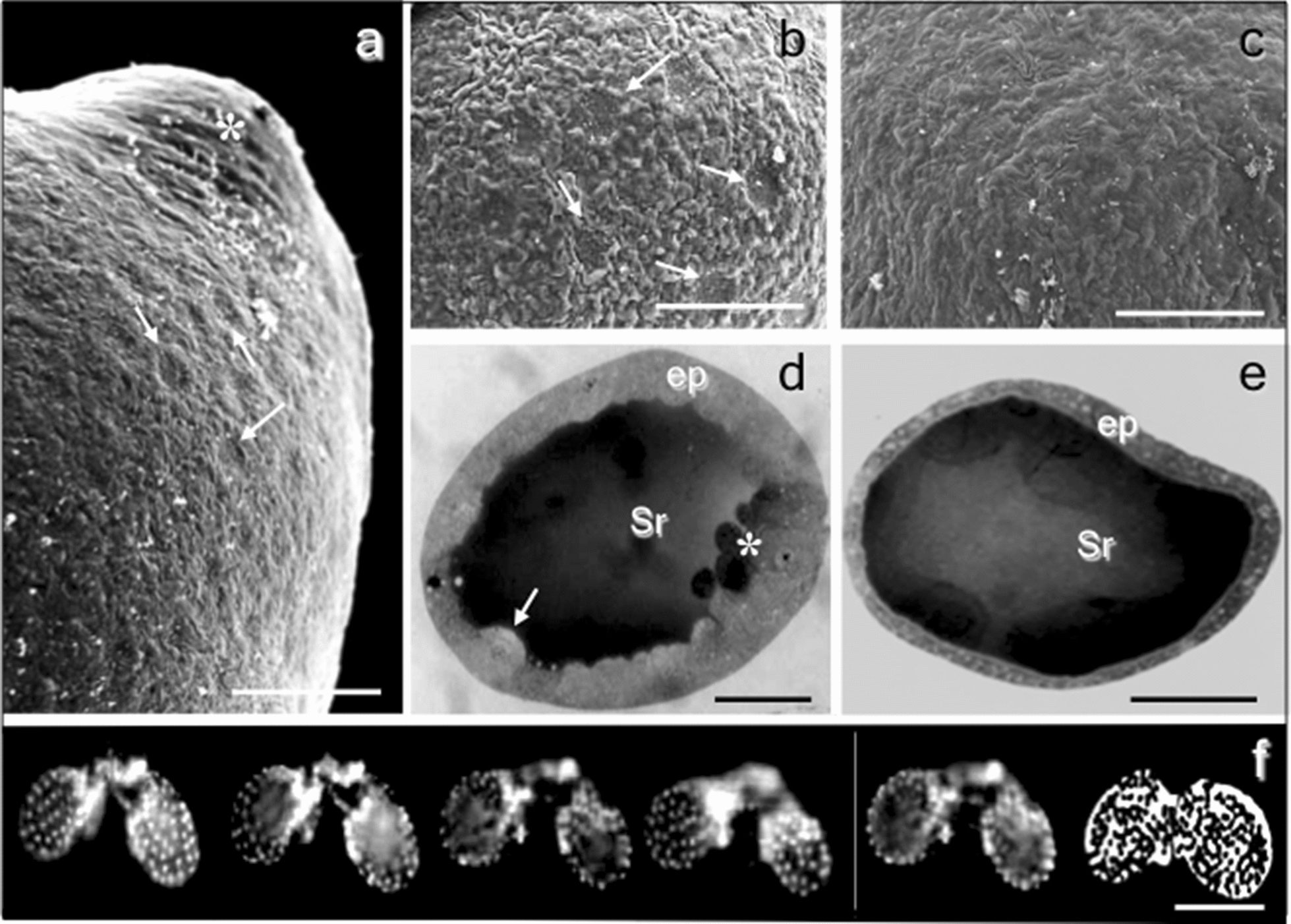
SEM micrographs showing enlarged images of sand fly SGs of *P. sergenti*. **a** and **b** show the marks of the epithelial cells (arrows) and **c** shows a shrunken surface of a flaccid SG lobe. **d**, **e** Histological sections of SGs, respectively, of *Lu. longipalpis* (**d**) and *Lu. migonei* (**e**). The secretory epithelium (ep) of *Lu. migonei* is in a straight line differing from *Lu. longipalpis*, which shows a single lucent cell (arrow) or a group of dark cells (asterisk) projecting into the saliva reservoir (Sr). **f** Sequential photomicrographs of SGs stained with the nuclear mark (DAPI) pictured at distinct focuses (from 16 images). All nuclei were marked over a transparent paper (as seen in the sketch on the right) and the cell number was counted. *Scale-bars*: **a, b, c**, 20 µm; **d**, **e**, 30 µm; **f**, 200 µm

The SEM revealed the surface topography of the exposed SGs of the five sand fly species displaying paired glands attached to the heads and each consisting of a pair of rounded lobes (Fig. 1a–d). Most of the SG lobes were turgid with marks on the surface of the internal secretory epithelial cells (Figs. 1c, 2a, b), but few SG lobes were totally (not shown) or partially flaccid with roughening (Fig. 1c) or shrunken surfaces (Fig. 2c). The same SG can display one turgid lobe and one flaccid lobe (Fig. 1c). In all sand fly species, each SG lobe is drained by the salivary duct, which join together to the common salivary duct (Fig. 1d).

The histology revealed SG lobes composed by a single epithelium surrounding the organ lumen, the saliva reservoir (Fig. 2d,e). Several types of secretory cells of distinct densities and varying from cylindrical to cubical shapes constituted SG epithelium. The saliva inside the lumen (saliva reservoir) presented a strong dark-staining feature (Fig. 2d, e). In some cases, the SG secretory epithelium was in a straight line, as observed in *Lu. migonei* (Fig. 1e), while in other cases, groups of basophilic epithelial cells and single lucent cells may be seen projecting into the lumen, as observed in *Lu. longipalpis* (Fig. 1d).

Using photomicrographs with sequential focuses of the entire SGs stained with the fluorescent nuclear marker DAPI, the total number of secretory cells was counted in each gland/lobe (Fig. 1f). There were 105 and 120 secretory cells in each lobe of *Lu. longipalpis* and *Lu. migonei*, respectively.

### The lectin-binding to SGs of sand fly species

The SGs of the distinct sand fly species diverged or had similar labelings of their structures according to the sugar-binding properties revealed by distinct fluorescent lectins when analyzed under LSM.

**Fig. 3:**
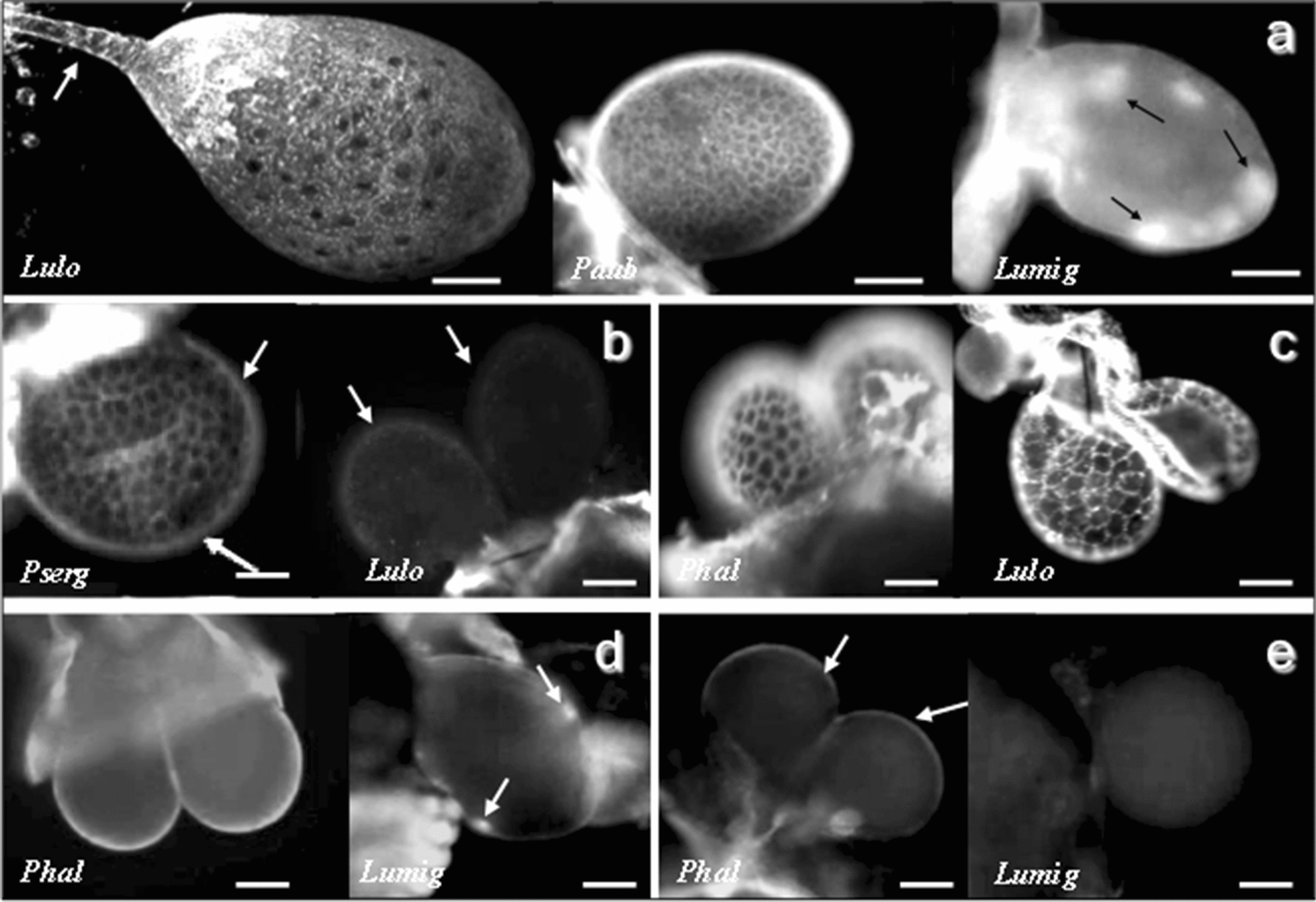
LSM pictures of lectins labeling the SG. **a** Con A lectin labeling of *Lu. longipalpis* (*Lulo*, a 3D image), *P. duboscqi* (*Pdub*) and *Lu. migonei* (*Lumig*). Note the presence of strong labeling in some secretory cells of *Lu. migonei* (arrows in *Lumig*). *Scale-bars*: *Lulo*, 40 µm; *Pdub* and *Lumig*, 50 µm. **b** HPA A lectin. The SG of *P sergenti* (*Pserg*) shows labeling in the basal lamina (arrows) and in the intercellular spaces of the secretory cells. In the SG of *Lu. longipalpis* (*Lulo*), the labeling is almost not visible (arrows). *Scale-bars*: *Pserg*, 50 µm; *Lulo*, 100 µm. **c** WGA lectin. The SGs show strong reactions in the intercellular spaces among the epithelial secretory cells in *P. halepensis* (*Phal*) and *Lu. longipalpis* (*Lulo*). *Scale-bars*: 100 µm. **d** RCA lectin. The SG is labeled in the basal lamina of *P. halepensis* (*Phal*) distinctly from *Lu. migonei* (*Lumig*), which presents a few fluorescent secretory cells. *Scale-bars*: *Phal* and *Lumig*, 100 µm. **e** UEA lectin. The SG of *P. halepensis* (*Phal*) is showing weak labeling in the basal lamina (arrows) differently from *Lu. migonei* (*Lumig*), which has a complete negative reaction. *Scale-bars*: 100 µm
(ii) HPA. The basal lamina of the SG of *P. sergenti* presented visible labeling distinct from the almost invisible labeling in *Lu. longipalpis* (Fig. 3b).
(iii) WGA. The intercellular spaces between all secretory cells showed strong lectin-binding in all SGs of the sand fly species, as shown for *P. halepensis* and *Lu. longipalpis* (Fig. 3c).
(iv) RCA. The SG basal lamina showed intense fluorescent labeling according to the observed species of sand fly,

(i) Con A. All SG secretory cells of most of the sand flies were fluorescently labeled as shown for *L. longipalpis* and *P. duboscqi* (Fig. 3a). The exception was the SGs of *Lu. migonei* which showed specific strong labeling of only a few secretory cells (Fig. 3a).

### Distinct types of secretory cells in sand fly SGs

**Fig. 4:**
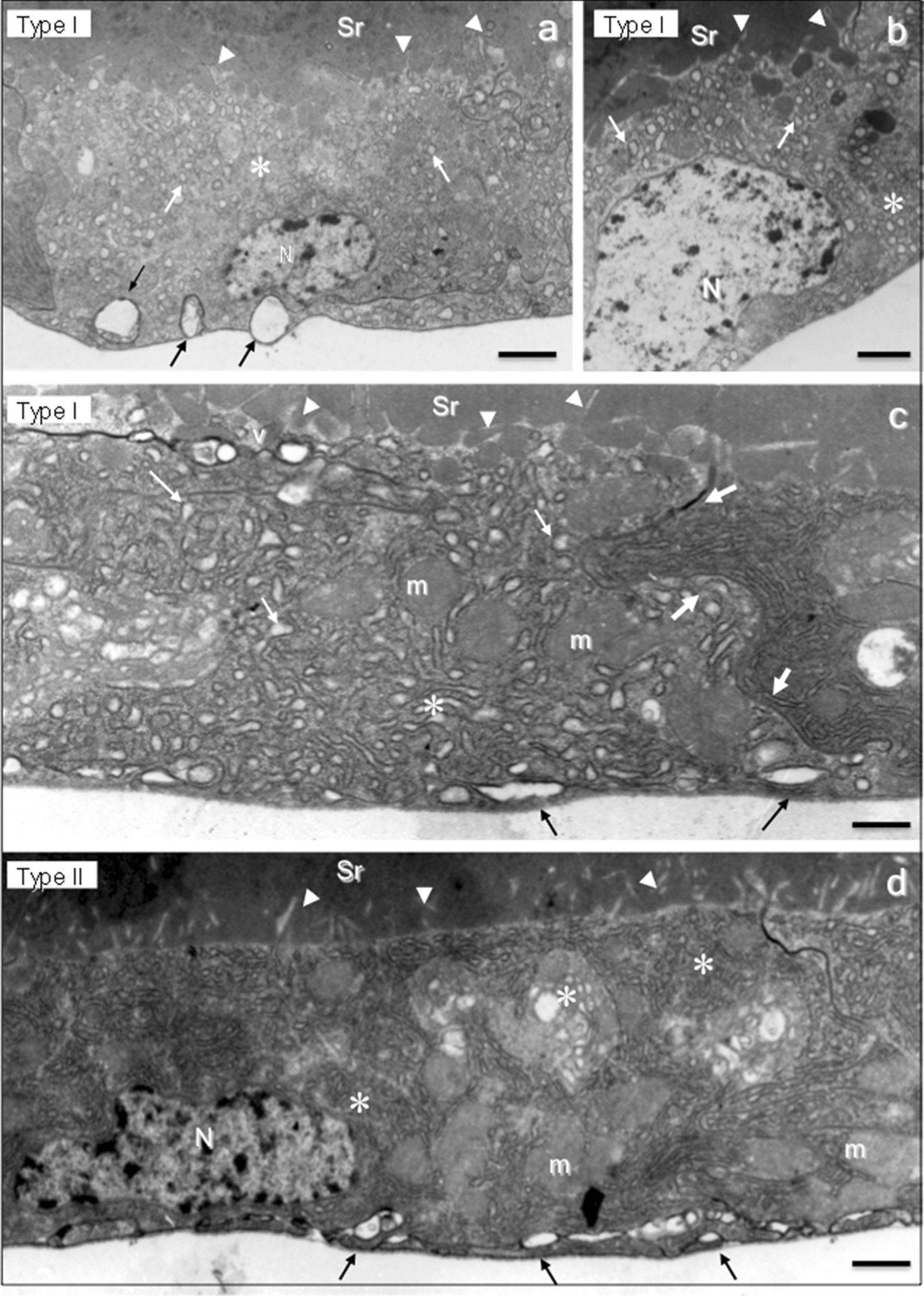
Ultrastructure of *Lu. longipalpis* SG. **a-c** Aspects of the secretory cell type I. Note the extensive and enlarged endoplasmic reticulum (asterisk) full of secretion and vesicles (white arrows). **c** The limit between two secretory cells (large white arrows). **d** A type II cell full of endoplasmic reticulum (not enlarged) without vesicles. Mitochondria (m) in the cytoplasm and several vesicles (black arrows) close to the basal lamina are seen in the type I cells (**a** and **c**). Cytoplasmic projections (arrowheads) are also seen in the cell surface of the two cell types. *Abbreviations*: Sr, saliva reservoir; N, nuclei. *Scale-bars*: **a**, 3 µm; **b**, 2.5 µm, **c**, **d**, 2 µm

**Fig. 5:**
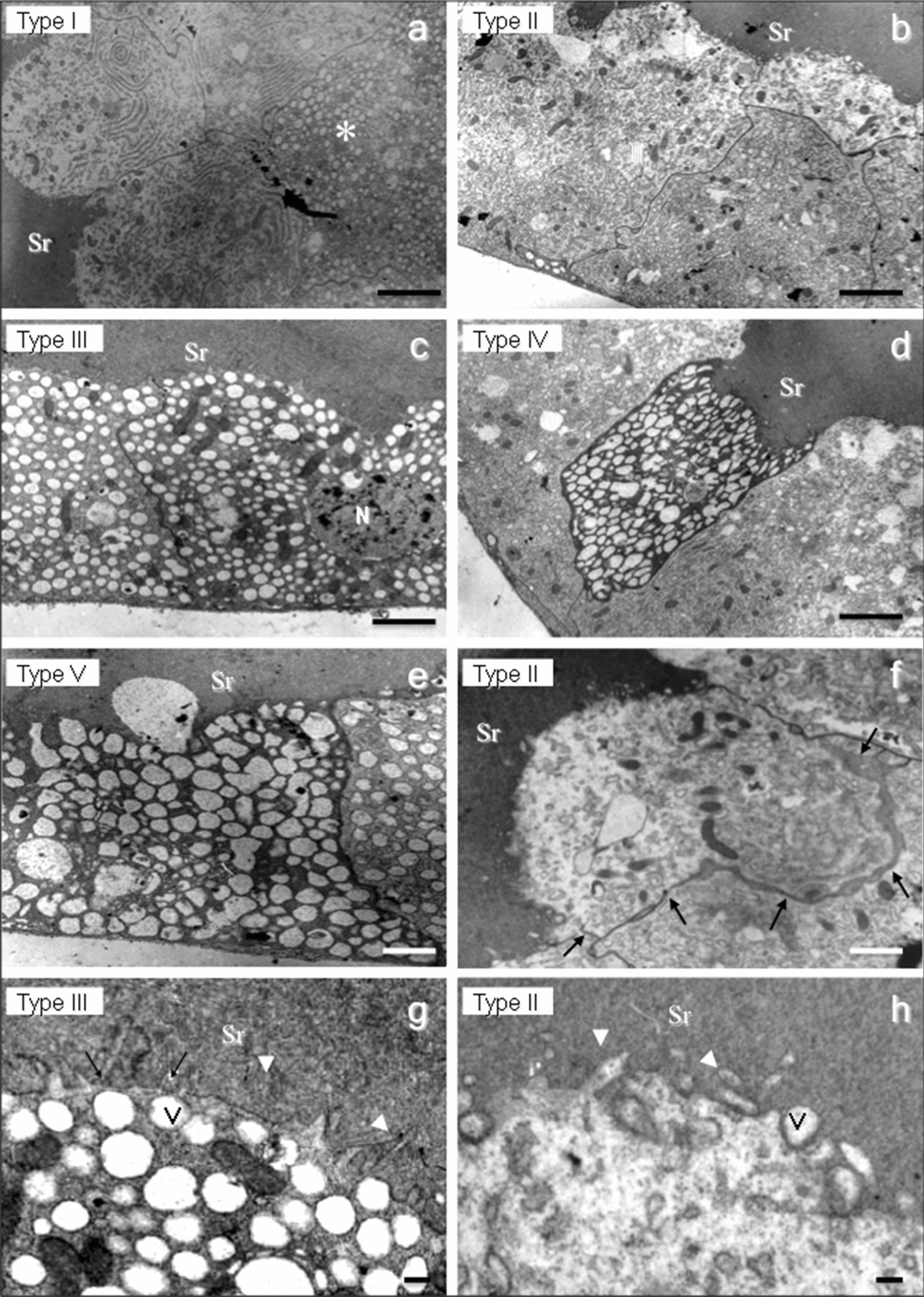
Ultrastructure of *Lu. migonei* SG. Panels **a** through **e** are showing, respectively, the cell types I through V. Details of two secretory type II cells separating from each are shown in panel **f** (arrows). Panels **g** and **h** are enlarged images of the secretory cell surfaces, respectively, of the cells types III and II, showing aspects of cytoplasmic projections on their surfaces (arrowheads) and secretory vesicles (V). *Scale-bars*: **a**, **b**, **e**, 2.5 µm; **c**, 2.0 µm; **d**, **f**, 1.5 µm; **g**, **h**, 1.0 µm

**Fig. 6:**
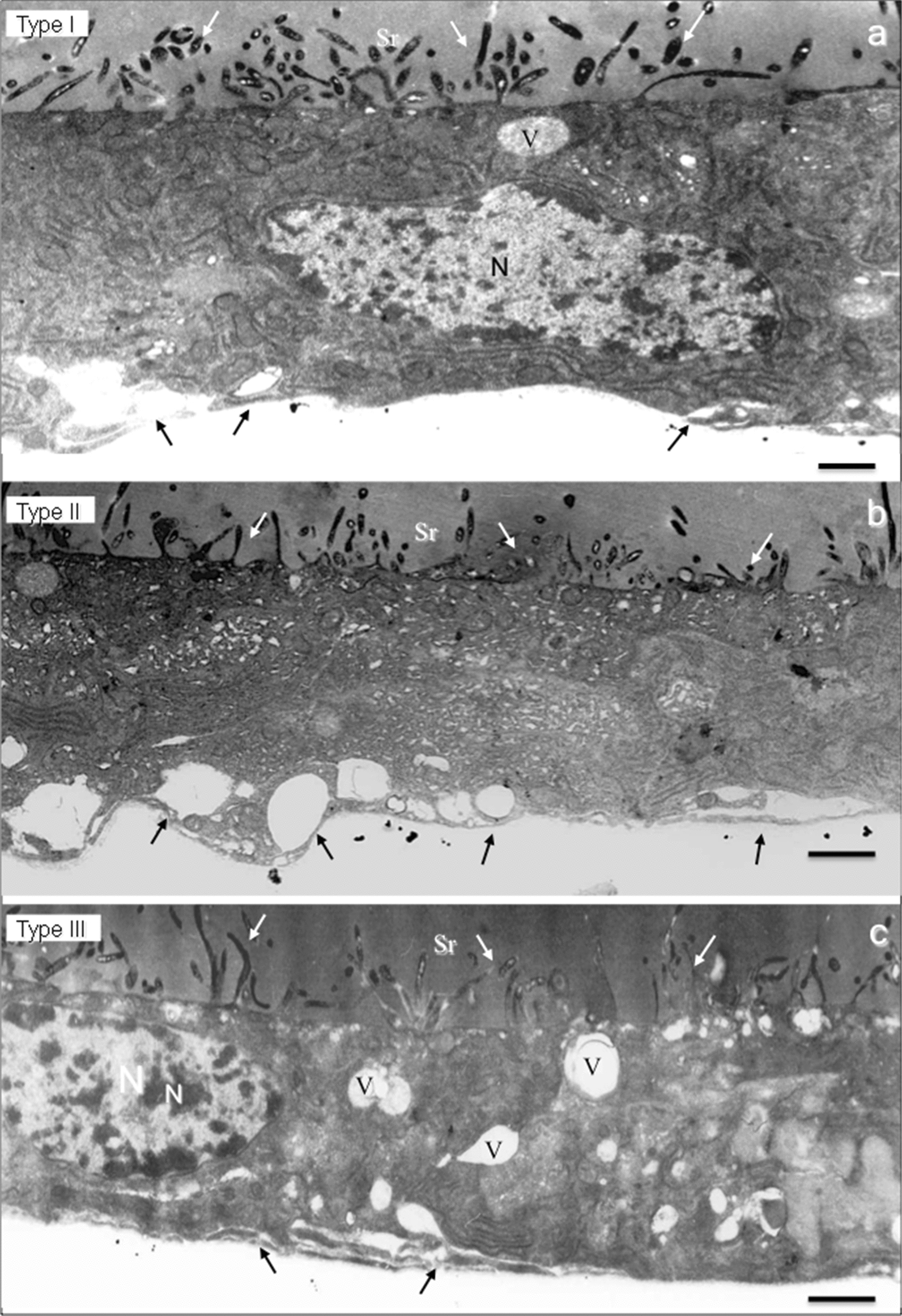
Ultrastructure of *P. duboscqi* SG showing the secretory cells, respectively, of type I (**a**), type II (**b**) and type III (**c**). Several vesicles are close to the basal lamina of all cell types (black arrows). Also, observe the aspect of the large cytoplasmic projections over the cell surfaces presenting a dark staining (white arrows). *Abbreviations*: Sr, saliva reservoir; N, nuclei; V, cytoplasmic vesicles. *Scale-bars*: 2.5 µm

The ultrastructure of the sand fly SGs analyzed by TEM showed different types of secretory cells comprising the single epithelium that surrounds the saliva reservoir. Most of the secretory cells were stretched cells and all of them with the apical surface in the direction of through the saliva reservoir (Figs. 4, 5, 6). They have a large oval single nucleus positioned in the basal cytoplasm close to the basal lamina. These nuclei have euchromatin and heterochromatin domains with the latter mainly localized as electron-dense patches in the periphery (Figs. 4a, b, d, 6a, c).

The cell cytoplasm of the sand fly SG secretory cells had different aspects varying in density and diverging in the number and shape of secretory vesicles, and amount of mitochondria and features of the endoplasmic reticulum. These ultrastructural characteristics varied according to the sand fly species.

The secretory cells of the sand fly SGs were classified into 5 different types according to the ultrastructural aspects of their cytoplasmic organelles: type I cells (with large amounts of endoplasmic reticulum cysterns and mitochondria; Figs. 4a-c, 5a, 6a); type II cells (with intense secretory activity presenting unorganized cytoplasm in the apical region with a massive presence of several secretory vesicles opening their contents inside the saliva reservoir; Figs. 4d, 5b, f, h, 6a); type III cells (with cellular cytoplasm filled with electron-lucent secretory vesicles of several sizes and few mitochondria; Figs. 5c, g, 6); type IV cells (with cell surface with scarce microvilli, numerous electron-lucent vesicles with distinct shapes and apparently with no or rare material inside; Fig. 5d); and type V cells (with highly electron-dense cellular cytoplasm with the presence of several large electron-lucent rounded vacuoles; Fig. 5e).

## Discussion

The SG of biting insects is responsible for synthesis and secretion of the saliva that is inoculated into the pierced skin of humans and animals. The saliva’s ability to commandeer the host’s hemostatic system has likely evolved to facilitate vector blood acquisition [10]. The importance of sand fly saliva to counteract the host’s hemostatic system has been extensively studied over the years (see [6] for a review).

On the host’s side, the first few days of the infection are critical for *Leishmania* survival. The recently transmitted parasite has to deal with the host’s immune response [22]. In this regard, substances of sand fly saliva enhance the infection when they are co-inoculated with *Leishmania* in the host [23–26]. Sand fly saliva antigens are capable of inducing delayed-type hypersensitivity in experimental hosts and humans [27, 28] and a specific antibody response [12]. Sand fly saliva also activates T cells and macrophages by inhibiting the expression of Th1 type cytokines and inducing the expression of Th2 cytokines [29], possesses chemotactic activity for macrophages, helping *Leishmania* to enter their target cells [30], inhibits dendritic cells antigen presentation capabilities, and increases apoptosis of neutrophils, major components in defense of fighting infections [31, 32]. The sand fly saliva also confers protection against *Leishmania* prior to exposure of mice to bites of uninfected sand flies [33]. To date, several proteins from different families have been identified in the saliva with several proteins being shared between *Lutzomyia* spp. and *Phlebotomus* spp. [34]. Several studies strongly suggest that sand fly saliva proteins are relevant for the epidemiology of leishmaniasis and can be a potential target for a vaccine against leishmaniasis [6, 33, 35, 36].

A comprehensive study of the sand fly SG structure, including defining its ultrastructural properties, is fundamental for helping to understand the biology and the physiology of synthesis and secretion. Our results revealed that the typical anatomy of the SGs of species of the genera *Lutzomyia* and *Phlebotomus* appears to be generally conserved, but with some distinction in the different species of sand flies.

We found no histological or ultrastructural differences between the two lobes observed in all studied sand fly species. In mosquito SG, it is well known that the secretory cells of the anterior part of the lobules are associated with sugar-feeding, while the cells of the posterior part are associated with blood-feeding [37, 38]. Differently, in the sand fly SG, no particular region related to a specific secretory pathway was found, though, our analysis only focused on sugar-fed sand flies. It seems that the two lobes produce the same saliva substances, which are stored in a saliva reservoir. However, we distinguished turgid and flaccid SG lobes, probably reflecting the difference of saliva contents or protein concentration. This fact indicates that the SG lobes of the sand flies may be stimulated to secrete saliva in distinct moments. In some insects, the production and secretion of the saliva occur at the same time, but, in insects with saliva reservoirs, the secretion is regulated [37]. Two types of regulation have been found: endocrinal and neuronal [39]. As an example, the SG of *Aedes aegypti* is surrounded by nervous complexes, which liberate serotonin controlling the secretion of the saliva [40]. In our study, no nervous complexes or muscle fibers on the surface of the SG were observed and further studies are necessary for better understand specificities of sand fly SG secretory pathways.

Interestingly, sand fly SGs are composed by a relatively small number of secretory cells, around 100–120 cells in each lobe. According to their ultrastructure we classified the secretory cells in five types. These morphological types of SG secretory cells were present with some dissimilarity among the species. Two types of secretory cells were found in *Lu. longipalpis* (type I and type II), three in *P. duboscqi* (type I, type II and type III) and all the five types of cells were found in *Lu. migonei*. The SG of *Lu. migonei* presented the greatest variety of the secretory cells with all the five different types. It is possible that some cellular types are immature cells still in development; however, we observed this variety of types of secretory cells completely differentiated (with few organelles and filled with secretory vesicles), suggesting that they are in fact subpopulations of distinct mature secretory cells.

It is remarkable that the sand fly SG is an organ composed of a small number of secretory cells with few different cell types, although they produce and secrete several substances to form the saliva. A proteomic approach has identified 20–40 proteins belonging to 13 protein families in distinct sand fly species [11, 35, 41, 42]. This fact suggests that each type of secretory cell might be involved in the production and secretion of different salivary components. In the present study, the morphology showed that in some SGs the epithelium is in a straight line while in others, groups of secretory cells are projected and appear to be released in the saliva reservoir. For example, according to the ultrastructural aspect of saliva secretion, cell types I and V are merocrine cells. Their secretory vesicles are excreted *via* exocytosis to the saliva reservoir, i.e. a process of transient vesicles fusing with the plasma membrane. However, cell types II and III are exocrine cells. Their saliva secretion is accompanied with loss of cytoplasm. Moreover, the type IV cells are holocrine cells. In this secretory process the entire cell is released from the secretory epithelium with all its contents resulting in the death of the secretory cell. The holocrine process is a programmed cell death, an apoptosis mechanism [43]. These findings reveal that the sand fly SG is a multifaceted exocrine gland with a variety of types of secretory cells, which execute a distinct secretory process of the saliva.

In the last decades, lectins have been employed in the detection of carbohydrates on insect salivary glands, such as *Aedes aegypti* [44], *Anopheles stephensi* and *Anopheles albimanus* mosquitoes [45], and *Glossina* spp. tsetse flies [46]. In *Ae. aegypti*, Con A lectin was useful for the recognition of the medial lobe [44], the site of penetration by sporozoites of *Plasmodium* [45]. Subsequently, similar data were reported for the *An. gambiae* complex [47]. In sand flies, specific reactions of Con A and WGA lectins revealed complex type of N-glycans in glycoproteins present in the saliva extract of *P. duboscqi* and *Lu. longipalpis* [12, 42]. Here, the lectin labeling showed that the SGs, according to the observed cellular structures or sand fly species, differ in some of their sugar epitopes. Curiously, there was no lectin labeling of any sand fly’s saliva resevoir, which could be due to the small lectin concentration or even the formaldehyde fixation process. However, we have effectively shown some shared and also specific sugar epitopes in the SGs of the sand fly species. The fluorescent lectins facilitated characterization of these differences among the distinct sand fly SGs. They were marked to a different degree by specific secretory cells and their intercellular spaces, and the basal lamina. Especially, Con A and RCA lectin labelings in *Lu. migonei* SG confirmed the existence of specific secretory cell populations.

## Conclusions

To the best of our knowledge, we demonstrated for the first time that secretory cells comprising the SGs of sand flies can be classified according to their ultrastructure and lectin-binding into five different subpopulations with varying secretory processes. Further studies are necessary to better understand the role of these different cell types and better relate them with the production and secretion of saliva substances, which have a fundamental role in the interaction of sand fly vectors with *Leishmania*.

## Data Availability

Data supporting the conclusions of this article are included within the article. The raw datasets used and analyzed during the present study are available from the corresponding author upon reasonable request.

## Abbreviations

SG: salivary gland
LSM: laser scanning confocal microscopy
SEM: scanning electron microscopy
TEM: transmission electron microscopy
DAPI: 4,6 diamidino-2-phenylindole
Con A: *Canavalia ensiformis*
RCA: *Ricinus communis*
WGA: *Triticum vulgaris*
HPA: *Helix pomatia*
UEA: *Ulex europaeus*
